# Supplementary material for: Astaxantin and Isoflavones Inhibit Benign Prostatic Hyperplasia in Rats by Reducing Oxidative Stress and Normalizing Ca/Mg Balance
Source: Plants (Basel). 2021 Dec 12;10(12):2735. doi: 10.3390/plants10122735 (PMC8704012; doi:10.3390/plants10122735)
Supplement: Supplementary file 1 [file plants-10-02735-s001.zip › plants-1511885-supplementary.pdf]

Table S1. Prostatic hyperplasia scoring system (units) for rats. Adopted from Scolnic et al. (1994) with modification.

Parameter	Variant	Units
Low-power magnification (×100)		
Lumen shape	regular	1
	villamentous	2
	papillary	3
	cribnform	5
Acinar shape	tubular	1
	branched	3
	irregular	5
Interacinar space	large	1
	moderarte	3
	back to back	5
Stroma	fine	1
	moderate	3
	fibrosis	5
High-power magnification (×200-400)		
Epithelial shape	flat / cyboidal	1
	cylindrical	3
	hexagonal	5
Nuclear shape	round	1
	small large	2

	irregular	5
Mitoses per field (×400)	absent	0
	infrequent (1-2)	2
	abundant (3-5)	5
	excessive (>6)	10
Number of epithelial layers	one	1
	several (2-3)	3
	multiple >5	5
Incidence	focal	3
	diffuse	5
Cell alignment	polar	1
	apolar	3
	penacinar	3
	piling up	3
	budding out	5
	isolated clusters	5
Inflammatory infiltration	none	0
	single cells	1
	small infiltartes	3
	huge infiltrates	5
Total		
